# A Framework for Inclusion of Unmodelled Contact Tasks Dynamics in Industrial Robotics

**DOI:** 10.3390/s22197650

**Published:** 2022-10-09

**Authors:** Zaviša Gordić, Kosta Jovanović

**Affiliations:** University of Belgrade, School of Electrical Engineering, 11000 Belgrade, Serbia

**Keywords:** industrial robots, Industry 4.0, contact task dynamics, sensor data fusion

## Abstract

This paper presents a method to include unmodeled dynamics of load or a robot’s end-effector into algorithms for collision detection or general understanding of a robot’s operation context. The approach relies on the application of a previously developed modification of the Dynamic Time Warping algorithm, as well as a universally applicable algorithm for identifying kinematic parameters. The entire process can be applied to arbitrary robot configuration, and it does not require identification of dynamic parameters. The paper addresses the two main categories of contact tasks with unmodelled dynamics, which are determined based on whether the external contact force has a consistent profile in the end effector or base coordinate. Conclusions for representative examples analysed in the paper are applicable to tasks such as load manipulation, press bending, and crimping for the first type of forces and applications such as drilling, screwdriving, snap-fit, bolting, and riveting assembly for the latter category. The results presented in the paper are based on realistic testing with measurements obtained from an industrial robot.

## 1. Introduction

With Industry 4.0 becoming a reality and even a standard in some applications and Industry 5.0 emerging on the horizon, the need for seamless integration and interoperability of production entities is ever increasing. A better understanding of the operating context of all entities, including industrial and collaborative robots as some of the most versatile and agile production components, is one of the essential requirements for achieving these goals.

Industry 4.0 has also brought changes to material and resource flow and challenged traditional layouts, in which production cells with industrial robots were fenced into relatively isolated islands with restricted access [1]. The need for constant optimization of production resources has, in many cases, led to the removal of barriers and fences to enable an unobstructed flow of material, mobile robots, or human workers. This has introduced a new level of dynamism to the working environment of industrial robots, which is something they were not designed for. Although a partial solution is offered in the form of collaborative robots, their performance and robustness are often not sufficient for environments where high efficiency and repeatability are required and where human presence or intervention is not frequent. Moreover, while there are fields where robotization was first made possible by the concepts of Industry 4.0, in most cases, the transition will be more of an evolution than a revolution, meaning that industrial robots will retain a strong presence in the foreseeable future [1,2,3].

Many manufacturers of industrial robots and related equipment have already reacted according to these trends. As a result, they are now offering different Industry 4.0 functionalities, connectivity, and simulation-related options, at least for their new and upcoming products [3]. Furthermore, related topics that have made undisputedly valuable contributions to novel industrial concepts are: collision detection and avoidance [4,5,6,7,8,9,10,11,12,13,14,15,16,17], physical human–robot interaction [18,19,20,21,22,23,24,25], kinesthetic guidance [19,20,26], motion planning, digital twins, augmented reality, and facilitated programming.

**Related works:** Contact tasks are by far the most common type of tasks in industrial robotics, regardless of whether they relate to load manipulation, assembly, or processing [2]. Despite that fact, there are still aspects that have not yet been addressed adequately for various reasons [17,27]. One of these aspects is inadequate attention to the forces that occur at the end effector during contact tasks. Numerous studies and reliable algorithms are dedicated to model- and non-model-based detection of physical contacts between the robot body and the surrounding environment or humans. On the contrary, the inclusion of task-related dynamics at the end effector during intentional contact in manufacturing tasks is rarely addressed. The reasons include difficulty or complexity of mathematical modelling of some contact forces [28,29,30], inadequate process-related know-how, and intentional or unintentional underestimation of the significance of specific forces or phenomena. In Industry 4.0 and the transition to human-centred Industry 5.0, where information is one of the cornerstones, failing to include task-related contact dynamics may have severe implications for reliability [31], efficiency, and safety, especially in shared workspaces [23,24,25].

In applications where monitoring of forces at the end effector is implemented, it is often resolved with the inclusion of force/torque sensors between the end effector and the robot flange [17]. However, such solutions are unsuitable for some applications due to the nature of the occurring forces, which may have adverse effect on the sensors’ reliability or longevity. More importantly, possible anomalies that occur in the kinematic chain between the robot’s base and its flange may remain undetected.

On the other hand, a solution developed for detecting physical human–robot interaction based on filtering the measurement signals according to their dynamics does not require additional sensors and could be adapted for this purpose [8]. However, while this solution undisputedly has its place in numerous applications, it relies on a plethora of information and expert knowledge needed for modelling, signal analysis and processing, and adaptations for each particular case.

Solutions based on non-model-based collision detection [9,12,13,14] intrinsically include the dynamics of the task itself, and their principles do indeed correspond to the requirements for detection and comparison of contact force profiles. However, the relative drawback of this approach is that it is limited to application in repetitive tasks, including identical or very similar motion to the motion during which the reference was recorded. While periodic repetitive tasks often involve identical movements of the robot, the needs of Industry 4.0 often require higher flexibility.

**Paper contribution:** The motivation for this paper is to offer a contribution related to contact task dynamics that can be applied in different fields. Developed primarily for application in comprehensive collision detection, the proposed approach can also be used for quality control, preventive maintenance, or a general understanding of the context of the robot’s operation. For all the mentioned fields, it is crucial to monitor the profiles of forces occurring during the task and compare them with their respective reference values.

The approach proposed in this paper relies on recently developed modifications of the Dynamic Time Warping algorithm [13] together with algorithms for automatic identification of kinematic parameters of an industrial robot [32,33,34,35,36] to enable a reliable and applicable solution for inclusion of intentional dynamics of a robot’s end effector. Using the robot’s kinematic model, it is possible to determine projection of intentional contact forces onto individual axes of the robot and comparing them with expected values enables distinction between phases of normal operation and erroneous states, including collisions, load manipulation anomalies, and improper alignment. From the implementation perspective, the algorithm has numerous advantages, as it is configuration-indifferent; it relies on measurements available from the robot motors such as joint coordinates, currents, or estimated torques; and it does so without requiring any additional sensory equipment.

**Paper organization:** Following the introduction in the Section 1, the Section 2 is devoted to describing the underlying idea based on the practical challenges and recent scientific contributions, whereas the Section 3 addresses implementation aspects of the proposed approach. The Section 4 presents and discusses results from offline testing of the algorithm with data recorded from the industrial robot while performing two representative types of contact tasks. The overall conclusion is offered in the Section 5, along with possible topics for future research.

## 2. Underlying Idea

Considerations presented in the introduction section form the basis on which the proposed approach and algorithm are conceived. The approach will be designed to be implementable without expert knowledge, to work without additional sensors, and to be platform-independent. To achieve these goals, it will rely on previously developed algorithms for the identification of kinematic parameters and, more importantly, for non-model-based collision detection. The most important contribution of the paper will be in the augmentation of the modified Dynamic Time Warping (mDTW) algorithm in the form of the inclusion of coefficients related to a kinematic model of the robot. In this way, an entirely new dimension to the algorithm is added, enabling it to respond to changes in spatial relation of robot joints and the effects that external contact forces have on them.

All research and procedures relevant to the realization and implementation of the proposed algorithm will be briefly overviewed to understand better the information required and the level of automation it is possible to achieve, both of which are important aspects from the Industry 4.0 perspective. However, the focus will be on the fusion of this information to enable an effective and reliable algorithm for the inclusion of contact task dynamics. To simplify the analysis, an industrial robot with six revolute joints is considered, being the most common configuration, but all conclusions are also valid for robots with prismatic joints.

The starting assumption is that when a robot is performing a task that does not involve contacts or changes in weight, values of all measurements that are available at each robot joint are in accordance with nominal values, i.e., can be considered to be known based on a nominal model of the robot or in some other shape or form. Contacts with the surroundings or changes in weight cause deviations from these nominal values. The idea underlying the proposed approach and algorithm is that these deviations reflect contact task dynamics and that their correct interpretation can lead to the implicit inclusion of the unmodelled forces and torques that appear during contact tasks.

In this paper, deviations recorded while performing one representative example of the contact task are referred to as a reference sequence. Deviations recorded during the operation of the robot stored in a sequence with first-in-first-out logic will be referred to as the measurement vector. The correct interpretation of the deviations in the measurement vector based on its comparison with the reference sequence can indicate whether the deviation originates from an expected, desired contact whose dynamics was not included in the nominal model or from some erroneous state or condition.

In industrial robotics, signals available at the level of robot joint usually include current values, joint angles, and in some cases torques or their estimated values. Previous research and analysis of the available signal properties [12,13,31] has observed that the measurements of currents and torque estimations are repeatable for similar movements, but that sampling is often inconsistent due to a lack of true parallel-processing capabilities.

Dynamic Time Warping (DTW) [37] is considered a reliable algorithm for comparison of signals with uneven sampling, while mDTW, as its modification, enables matching the measurement vector to a segment of the reference sequence as well as implementation in real time [38]. It also has the advantage of implicitly including all dynamic events and phenomena without modelling or deep understanding, since it observes joint events, which are consequences of task dynamics. However, the drawback of mDTW is that it was designed to perform matching of signals while the robot performs the movement in an identical or very similar way, maintaining a similar joint posture. Periodic tasks that include such movement are very common, but in the general case and especially in Industry 4.0 applications, a robot is expected to be more agile and adaptable to meet the needs of the interconnected event-driven production environment. These requirements also relate to its movement, meaning that the straightforward implementation of mDTW is no longer suitable in the general case, since the movements for the same type of task may require significant changes in the spatial relation of the robot’s joints. Differences in joint spatial relation dictate differences in effects that contact forces have on individual joints, resulting in the different profiles of deviations that are noticeable on a joint level. Supplementing mDTW with values related to the kinematic model of the robot aims to solve these drawbacks.

## 3. Kinematically Augmented Modified Dynamic Time Warping

The first step in the inclusion of kinematics-related parameters into modified Dynamic Time Warping (mDTW) is a determination of which parameters would be most effective in reflecting the effects of the changes in spatial relation of robot joints, which affect the projection of external forces. The projection of external forces originating from the contact task onto axes of interest can be determined reliably using a kinematic model of the robot. For this reason, it is necessary to identify the kinematic parameters of the robot itself as well of its tool, i.e., end effector. Denavit–Hartenberg (DH) notation is one method that is commonly used for this purpose and, as such, is considered for the representation of the kinematic parameters in this paper and the algorithm alike. Still, the idea is also valid for any other notation. The DH model in this paper is represented in the form of homogenous transformation matrices H.

Starting from identifying robot DH parameters from the base of the robot to the flange to which the end effector can be attached, it is essential to note that it can be performed in numerous ways, including manual calculations. The general approach in this paper is to facilitate the implementation of the algorithm and reduce the chances of errors wherever possible using automated procedures. Previous research [34] has described that an automatic procedure that moves the individual axis while monitoring the position of the point of interest at the end effector can perform the identification of kinematic DH parameters of the robot. The procedure can effectively extract relevant parameters for rotational and prismatic joints alike, and therefore the configuration of the robot does not impose any applicability limitation. A similar principle was later further elaborated [35,36] with considerations regarding accuracy and tools that can be used for its successful implementation in realistic environments.

The benefit of using the aforementioned approaches is that they are not restrictive regarding the type of sensors that are used to determine the position of the observed point in some external reference frame. The position can be measured directly as long as the accuracy and measurement volume are not issues. A theodolite or any other measurement sensor can be used. Another approach is to measure the position indirectly, using joint encoder measurements and a built-in kinematics model all robots use for direct kinematic tasks. In this paper, the kinematic model was calculated using the coordinates of the TCP in the robot’s base frame, which were provided by the robot’s controller based on the measurements of the joint encoders. Common to all the mentioned identification approaches is that the DH parameters are calculated based on the trajectories that the observed point of interest has while the robot moves each of its axes individually. In the case of rotational joints, the trajectory will be circular, and the centre and orientation of the path will indicate the direction of the *z* axis of the observed current joint. From the formed spatial directions of *z* axes for each joint, directions of other axes can be calculated as well. In the case of non-parallel *z* joint axes, the common normal from the previous towards the current direction of the *z* axis will determine the direction of the new *x* axis, and the point of its intersection with the current *z* axis will determine the current coordinate origin. In the case of parallel *z* axes, the direction of the *x* axis is determined using identical rules. However, since the number of the common normal is infinite, any convenient point can be chosen as the current joint coordinate system origin. The *y* coordinate axis is determined to complete the right-handed Cartesian frame, forming the joint coordinate frame. The spatial relation of all joint coordinate frames can then be used conventionally to determine kinematic parameters in DH or any other notation. The entire identification process itself and the related calculations are described in detail in the aforementioned papers and as such are not elaborated in this paper.

Another aspect required for the completion of the kinematic model is the identification of DH parameters of the end effector, i.e., tool. For the purpose of automatic parameter identification, the TCP parameters can be determined using a procedure based on the analysis of images of the tool from two orthogonal planes [32,33], since the solution can deliver parameters directly in the form of a homogenous transformation matrix, making it easier to include in the overall model. However, any other procedure can be used as well.

Once the DH parameters of the entire robot and end effector have been obtained, it is possible to use them to determine projections of the contact force profile onto the individual joint of the robot. To that end, it is first necessary to determine the relationship between the joint of interest and the end effector frame. This is achieved by multiplication of matrices $H_{i-1}^{i}$ from the observed joint *o* to the end effector indexed with *e* (1) and (2):

(1) $$H_{i-1}^{i}=\left[\begin{array}{cc} \begin{array}{c} \cos\left(\Theta_{i}\right)\\ \begin{array}{c} \sin\left(\Theta_{i}\right)\\ 0\\ 0 \end{array} \end{array} & \begin{array}{ccc} \begin{array}{c} -\sin\left(\Theta_{i}\right)\cos\left(\alpha_{i}\right)\\ \begin{array}{c} \cos\left(\Theta_{i}\right)\cos\left(\alpha_{i}\right)\\ \sin\left(\alpha_{i}\right)\\ 0 \end{array} \end{array} & \begin{array}{c} \sin\left(\Theta_{i}\right)\sin\left(\alpha_{i}\right)\\ \begin{array}{c} -\cos\left(\Theta_{i}\right)\sin\left(\alpha_{i}\right)\\ \cos\left(\alpha_{i}\right)\\ 0 \end{array} \end{array} & \begin{array}{c} a_{i}\cos\left(\Theta_{i}\right)\\ \begin{array}{c} a_{i}\sin\left(\Theta_{i}\right)\\ d_{i}\\ 1 \end{array} \end{array} \end{array} \end{array}\right]$$

(2) $$H_{o}^{e}=\overset{e}{\underset{i=o}{\prod }}H_{i}.$$

Based on the calculated matrix $H_{o}^{e}$, it is possible to determine the distance between joint of interest and the Tool Centre Point (TCP) in coordinate frame of the observed joint of interest *o* (3):

(3) $$\left(x_{oe},y_{oe},z_{oe}\right)=\left(H_{o}^{e}\left(1,4\right),H_{o}^{e}\left(2,4\right),H_{o}^{e}\left(3,4\right)\right)$$

**Figure 1:**
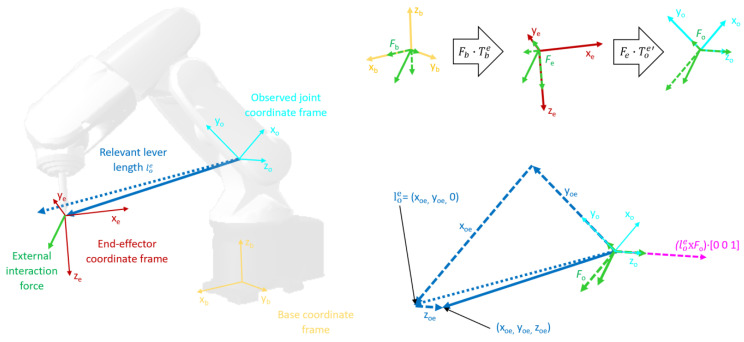
Coordinate frames and transformations. The left section of the figure illustrates the base and tool coordinate frame as well as the coordinate frame of the observed joint together with levers and direction of the external interaction force. The upper right section illustrates the steps of transforming the external force into the coordinate frame of the observed joint. The lower right section of the figure shows the distance between the origins of coordinate frames of the observed joint and the end effector projected onto the x-y plane to determine the relevant lever length. This section of the figure also shows the relevant component of the torque, whose absolute value equates to the equivalent lever length.

The calculated distances in the relevant joint coordinate frame effectively represent the lever lengths that may affect the torque in that joint. Since for single-degree-of-freedom (DoF) revolute joints the only axis affected by torques is the *z* axis, the actual lever components that may affect the torque are those perpendicular to the *z* axis. This means that only x and y components of the lever are relevant for further calculations, and therefore, they are the only ones to be selected (4), as depicted in Figure 1 in blue.

(4) $$l_{o}^{e}=\left(x_{oe},y_{oe},0\right)$$

If the normalized components of contact force are known in the robot base coordinate frame, $F_{b}$, they first need to be transformed into the coordinate frame of the tool (5)–(7):

(5) $$H_{b}^{e}=\overset{e}{\underset{i=1}{\prod }}H_{i-1}^{i}$$

(6) $$T_{b}^{e}=\left[\begin{array}{ccc} H_{b}^{e}\left(1,1\right) & H_{b}^{e}\left(1,2\right) & H_{b}^{e}\left(1,3\right)\\ H_{b}^{e}\left(2,1\right) & H_{b}^{e}\left(2,2\right) & H_{b}^{e}\left(2,3\right)\\ H_{b}^{e}\left(3,1\right) & H_{b}^{e}\left(3,2\right) & H_{b}^{e}\left(3,3\right) \end{array}\right]$$

(7) $$F_{e}=F_{b}\cdot T_{b}^{e}$$

where $H_{b}^{e}$ is the homogenous transformation matrix from the coordinate frame of the robot base into the coordinate frame of the tool, and $F_{e}$ represents components of the force in the end-effector coordinate frame, as shown in the upper right part of Figure 1 in green colour.

If the normalized components of the contact force $F_{e}$ are known in the coordinate frame of the tool, steps (5)–(7) can be skipped. Once the relevant kinematic chain has been calculated, it is then possible to determine its components in the coordinate frame of the joint of interest (8) and (9).

(8) $$T_{o}^{e}=\left[\begin{array}{ccc} H_{o}^{e}\left(1,1\right) & H_{o}^{e}\left(1,2\right) & H_{o}^{e}\left(1,3\right)\\ H_{o}^{e}\left(2,1\right) & H_{o}^{e}\left(2,2\right) & H_{o}^{e}\left(2,3\right)\\ H_{o}^{e}\left(3,1\right) & H_{o}^{e}\left(3,2\right) & H_{o}^{e}\left(3,3\right) \end{array}\right]$$

(9) $$F_{o}=F_{e}\cdot {T_{o}^{e}}^{\prime }$$

The cross product of relevant lever length components and components of the contact force in the tool coordinate frame yields components of torques in the tool coordinate frame, while the absolute value of the *z* component of the cross product, shown in purple in Figure 1, is equal to the equivalent lever length for the joint of interest (10).

(10) $$l_{eqiv}=\left|\left(l_{o}^{e}\times F_{o}\right)\;\left[0\;0\;1\right]\right|$$

Calculation of the lever lengths for each sample contained in the measurement vector and reference signal enables morphing both the reference value and the measurements to enable better comparison. When values of the measured samples, i.e., deviations from the nominal values, and corresponding lever lengths are observed, it is evident that there is a significant correlation. Depending on the dynamics of the measurement signal caused by the robot’s movement on the observed sequence, type of task, and observed joint, this correlation coefficient ρ∈[0,1] ranges from 0.64 to 0.95, while a simple smoothing filter raises the lower limit to 0.84, confirming the intuitive assumption.

At this point, it is important to make a physical interpretation of the connection between the calculated equivalent lever lengths and the deviations in the measurement vector. Deviations within the measurement vector correspond to torques produced by the unmodelled external contact force acting on the equivalent lever length. The intensity of force components does not have to be known explicitly, since the effect of components on an individual joint axis is implicitly reflected through intensities of deviations.

From the implementation perspective, to enable calculation of the lever lengths, the measurement signal received from the robot must contain measurements of joint angles, in addition to the measurements of joint currents or estimated torques, which are used to indicate the presence of contact. For each joint, the measurement vector $M_{v}\left(i\right)$ contains two pieces of information, joint current or estimated torque a(i) and rotation angle from the encoder $\Theta_{v}\left(i\right)$. Similarly, measurements based on which the reference sequence $M_{r}\left(j\right)$ was stored contain components of joint current or estimated torque *b*(*j*) and rotation angle $\Theta_{r}\left(j\right)$.

(11) $$M_{v}\left(i\right)=\left(a\left(i\right),\Theta_{v}\left(i\right)\right),\;1<i<m$$

(12) $$M_{r}\left(j\right)=\left(b\left(j\right),\Theta_{r}\left(j\right)\right),\;1<j<n$$

To begin with a short overview, DTW is a well-known method and is considered a reference for shape-based matching of the signals in time series. It allows contraction and/or dilatation of a signal in the time domain using matrices to find sequences of best-matching pairs of samples from signal *a* containing m samples and *b*, which consists of n samples. This is done using matrix $d_{mxn}$, formed according to predefined rules and based on which optimal pairs are determined. To achieve a minimal absolute difference, any sample of signal a can be matched with multiple samples of signal b, as well as the opposite, which effectively causes apparent signal contraction or dilatation in the time domain. Best-matching pairs within the matrix d are found using predetermined rules for the search, which starts from the last sample of both sequences d(m,n) and stops when the algorithm has reached the first samples of both signals d(1,1). The search does not allow going back, preserving the order of samples and, therefore, the causality in matching. However, starting and ending points of the search algorithm imply that DTW can only be effectively used to compare signals with similar content. To facilitate better understanding and comparison, the notation used in this paper is consistent with that in the original papers. To avoid any confusion or misinterpretation, symbol a used in the DTW algorithm corresponds to a signal that is compared and has no relation to the kinematic parameter a in DH notation.

Modified DTW (mDTW) changes some of the rules of DTW used to form the matrix d (13)–(16).

(13) $$\begin{array}{cc} d\left(1,j\right) & =\left|a\left(1\right)-b\left(j\right)\right|\\ & \;+min\left(\left|a\left(2\right)-b\left(j+1\right)\right|,\left|a\left(2\right)-b\left(j\right)\right|,\left|a\left(1\right)-b\left(j+1\right)\right|\right),\\ & \;i=1,\;j\neq n \end{array}$$

(14) d(1,n)=|a(1)−b(n)|+|a(2)−b(n)|,i=1, j=n

(15) d(i,1)=|a(i)−b(1)|+d(i−1,1),1<i≤m, j=1

(16) $$\begin{array}{cc} d\left(i,j\right)= & \left|a\left(i\right)-b\left(j\right)\right|+min\left(d\left(i-1,j-1\right),d\left(i,j-1\right),d\left(i-1,j\right)\right),\\ & \;1<i\leq m,\;1<j\leq n \end{array}$$

Compared to DTW, the difference is that Rule (13) implies that all elements in the first row of matrix d are calculated based on the absolute difference between the first sample of signal a, a(1) and the *j*-th sample of signal b(j) increased by a minimal absolute difference of any of the neighboring succeeding samples. This ensures that causality is preserved as well as that each of the elements in the first row can potentially be the end point of the search. This means that the search no longer has to end with d(1,1) but rather can end with any element in the first row, enabling signal a, which supposedly corresponds to a certain subsequence of signal b, to start at any sample of signal b. Rule (14) is a special case of Rule (13), which ensures the consistency of the search algorithm. Rules (15) and (16) are the same as in the original DTW, and they ensure causality.

Another difference in mDTW is that the search for optimal pairs starts from the element with minimal value in the last row of the matrix, min(d(m,j)), 1 ≤ *j* ≤ *n*, and not necessarily d(m,n). The search continues looking for the minimum of preceding values, as described with (17) and (18), and stops when the first row of the matrix d is reached.

(17) min(d(i−1,j−1),d(i,j−1),d(i−1,j)),i≠1, j≠1

(18) d(i−1,j),i≠1, j=1

Values increase monotonically with an increase in row and/or column number, inherently ensuring that the minimal value in the last row is the optimal starting point for the search. Modifications (13)–(16) ensure that each field, with the exception of those in the first row, includes information about the minimal sum of deviations before it, making sure that the optimal path to that particular point can be traced back to the first row. These rules enable mDTW to find the optimal pairs between signal a and best-matching subsequence of signal b. Applied to the case of the algorithm proposed in this paper, the measurement vector would be optimally matched with the most similar subsequence of the reference sequence.

Inclusion of lever lengths into the mDTW is done with the substitution of Rules (13)–(16) with Rules (19)–(22).

(19) $$d\left(1,j\right)=\left|\frac{a\left(1\right)}{l_{a}\left(1\right)}-\frac{b\left(j\right)}{l_{b}\left(1\right)}\right|+min\left(\left|\frac{a\left(2\right)}{l_{a}\left(2\right)}-\frac{b\left(j+1\right)}{l_{b}\left(j+1\right)}\right|,\left|\frac{a\left(2\right)}{l_{a}\left(2\right)}-\frac{b\left(j\right)}{l_{b}\left(j\right)}\right|,\left|\frac{a\left(1\right)}{l_{a}\left(1\right)}-\frac{b\left(j+1\right)}{l_{b}\left(j+11\right)}\right|\right),i=1,\;j\neq n$$

(20) $$d\left(1,n\right)=\left|\frac{a\left(1\right)}{l_{a}\left(1\right)}-\frac{b\left(n\right)}{l_{b}\left(n\right)}\right|+\left|\frac{a\left(2\right)}{l_{a}\left(2\right)}-\frac{b\left(n\right)}{l_{b}\left(n\right)}\right|,i=1,\;j=n$$

(21) $$d\left(i,1\right)=\left|\frac{a\left(i\right)}{l_{a}\left(i\right)}-\frac{b\left(1\right)}{l_{b}\left(1\right)}\right|+d\left(i-1,1\right),1<i\leq m,\;j=1$$

(22) $$d\left(i,j\right)=\left|\frac{a\left(i\right)}{l_{a}\left(i\right)}-\frac{b\left(j\right)}{l_{b}\left(j\right)}\right|+min\left(d\left(i-1,j-1\right),d\left(i,j-1\right),d\left(i-1,j\right)\right),1<i\leq m,\;1<j\leq n$$

Within these rules, $l_{a}\left(i\right)$, 1<i≤m represents the equivalent lever length calculated for *i*-th sample of the measurement vector using $\Theta_{v}\left(i\right)$, whereas $l_{b}\left(j\right)$, 1<j≤n represents the equivalent lever length calculated for *j*-th element of the reference sequence using angle $\Theta_{r}\left(j\right)$, for the observed joint using (1)–(10). Effectively, while forming the matrix d, each of elements a(i) and b(j) are divided by their corresponding lever lengths calculated for the observed joint based on the measurements of the joint angles, whose values are stored as part of the measurement vector and reference sequence, respectively. This modification does not affect the causality, since the manner in which the elements of matrix d are formed is unchanged. The search algorithm can still end at any element in the first row, although it may no longer be the same element as in the mDTW.

Rules (23) and (24) for searching the optimal path in matrix d are the same as Rules (17) and (18) of the mDTW:

(23) min(d(i−1,j−1),d(i,j−1),d(i−1,j)),i≠1, j≠1

(24) d(i−1,j),i≠1, j=1

The search will start from the element in the last row that has the minimal sum, min(d(m,j)), 1 ≤ *j* ≤ *n*, though again, it may not be the same element as with the mDTW. Since a monotonical increase in value with an increase in row and/or column number is preserved, the starting element chosen in this way will surely be the optimal one for the start of the search. As with mDTW, finding the optimal path is ensured by the fact that elements in each row, with the exception of the first one, inherently point to the optimal preceding element, since other candidates surrounding it will have higher values.

To enable easier implementation and reduce the computational time and effort, division of reference signal sample b(j) with its corresponding lever length $l_{b}\left(j\right)$ does not have to be performed in each cycle of the comparison. Instead, all samples from the reference sequence can be divided with their corresponding lever lengths by piecewise division prior to the start of computation of the matrix d. This alteration has a considerable effect on the speed and number of calculations, since the reference sequence is significantly longer than the measurement vector. Similarly, since measurement vector *M_v_* has first-in-first-out logic, at each comparison cycle, calculation of a(i) and $l_{a}\left(i\right)$ must be performed only for the newest sample *m* using $M_{v}\left(a\left(n\right),\Theta_{v}\left(n\right)\right)$, while other values can be reused.

## 4. Testing Results—Contact Tasks

The type of contact tasks addressed in this paper are primarily so-called force contact tasks [28], in which both force and position need to be considered for successful execution. Here, they are further divided into two categories based on the direction of forces that are affecting the robot at the end effector side.

The first category includes tasks where the direction or profile of the external force is consistent in the robot’s base frame, and it does not change regardless of the configuration of the robot. The most obvious example and the one that has the biggest impact on the quality of anomaly detection due to its frequency of application is the weight during load manipulation tasks. Other examples or variations of load manipulation include operations such as tending the press for bending, stamping, or clinching operations, in which the workpiece needs to be held throughout the process.

In the second group of tasks, the direction of external force is relative to the orientation of the end effector, i.e., considered consistent in the tool coordinate frame. These assignments mostly include processing and assembly tasks where the robot or its end effector are used to exert some force to the external object. Examples from this group include tasks such as drilling, screwdriving, riveting, nailing, stapling, snap-fitting, and bolting.

It is important to note that there are tasks that are a combination of the mentioned types. These tasks, however, are not the topic of this paper, since, in most cases, they can be separated into more basic operations to which the classification and related conclusions can be applied.

**Figure 2:**
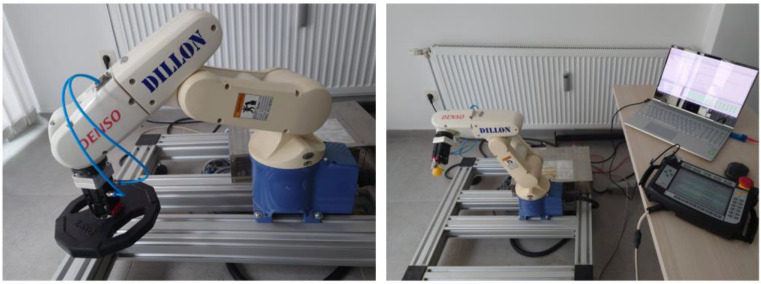
Measurement setups. (**left**) Load manipulation contact task. (**right**) Snap-fit assembly contact task.

For testing purposes, all data presented in this paper were recorded using Denso VP-6242 six-axis industrial robot while it was performing its tasks, as shown in Figure 2. Testing of algorithms was done offline on a PC but with real measurement data from the robot, recorded using data exchange between the PC and the robot’s controller. In a realistic scenario, data gathering and processing would be implemented in a similar way, and therefore, the testing results shown in this paper are considered to closely resemble the results that would be obtained with hardware-in-loop testing. However, it is important to mention that data exchange and processing can be implemented on other platforms to achieve better performance or convenience [39].

### 4.1. Consistent Base Frame Direction of Force

Following the general description of tasks within this category and relevant case of lever length calculations, this section aims to illustrate the inclusion and interpretation of contact task forces with consistent direction in the robot’s base frame while performing a representative task example. Load manipulation is one of the typical tasks from this category and perhaps the most intuitive to understand, since gravity is universally familiar.

To emulate possession of the optimal robot model, the first sequence of the movement was made without the presence of the load. The measurement from this movement represents an optimal model of the robot in which load dynamics are not included. The second sequence was recorded with actual load in a task where the robot simulates manipulation, in which the picking position is constant while placing positions differ one from the other. The first four placing positions feature identical orientations of the tool but with different distances from the base of the robot. The last two placing positions have identical tool orientations between them but differ compared to the first four and differ from each other in distances from the robot’s base. Absolute values of joint currents were used in the manipulation contact task, together with joint angles, to complete the measurement vector.

**Figure 3:**
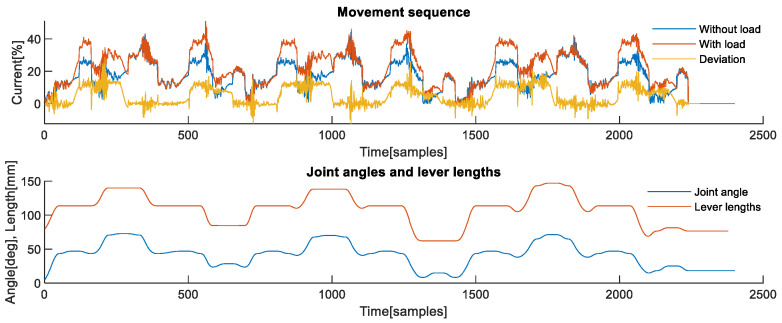
Signals obtained during the manipulation task from the 2nd axis. (**Upper**) Measurements of joint currents while the robot was performing the task without load and with the load. The difference between the two signals represented corresponds to the deviation profile originating from unmodelled dynamics of the contact task. Samples of deviation signal from each axis are one of the inputs to the proposed algorithm. (**Lower**) Measurement of the joint angle is one of the inputs to the proposed algorithm. The algorithm calculates equivalent lever lengths in each sample based on DH parameters and joint angles from all axes.

**Second axis analysis**: The analysis of the interaction effects of the manipulation task is best illustrated with the example of the second axis, since it is affected by the weight of the load due to its orientation. Figure 3 shows one complete sequence of six picking and six placing operations. The upper part shows signals that correspond to the sequence recorded without load, shown in blue, a sequence recorded with actual load, shown in red, and the difference between them, shown in yellow. The difference between the first two signals is the actual deviation caused by the weight of the load, i.e., deviation caused by the contact task force, which was not included in the nominal dynamic model of the robot. The lower part of Figure 3 shows joint angles of the observed second axis, shown in blue, which are used to calculate lever lengths shown in red colour. When lever lengths are observed in parallel with deviations caused by the weight of the load, their effect on the profile of the deviation is evident. Deviation signal along with the joint angles form the measurement.

**Figure 4:**
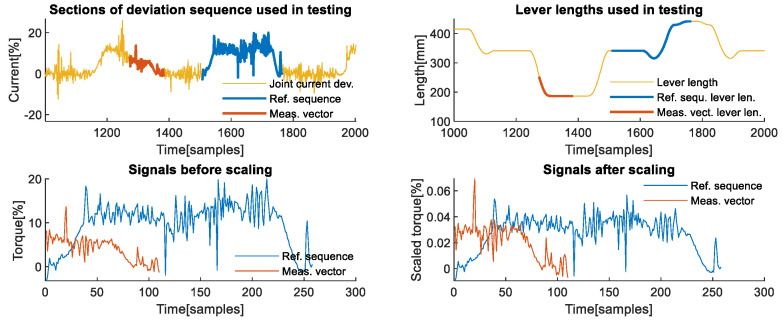
Signals used in the analysis for the 2nd axis. (**Upper left**) Sections of the deviation signal are used as a reference sequence and as a measurement vector during analysis. (**Upper right**) Highlighted sections of the lever lengths corresponds to the chosen reference sequence and chosen measurement vector. (**Lower left**) Original values of signals used as reference sequence and measurement vector shown prior to scaling by the algorithm. (**Lower right**) Signals used as reference sequence and measurement vector after scaling was performed.

The upper left part of Figure 4 shows the two sections of the deviations recorded on the second axis, which were used as examples of reference sequence and measurement vector for testing the algorithm’s effectiveness. Both the reference sequence, shown in red, and the measurement vector, shown in yellow, were chosen due to their differences in shape caused primarily by the different direction and intensity of change in their corresponding lever lengths, shown in corresponding colours on the upper right part of Figure 4. One important aspect when choosing the section that represents the reference sequence is that it needs to correspond to all phases that a contact task may have when the lever length is different from zero. In the case of manipulation contact tasks, it must include phases prior to picking up the load, lifting, transporting, lowering, releasing, and a brief section after the load is released. The lower left graph shows that the signals have different scales and that there is no obvious section of the reference sequence, shown in blue, that could reasonably be matched with the measurement vector shown in red. The lower right section of the same figure illustrates that after scaling, it is evident that, aside from the shift in time, there is a section of the scaled reference sequence that is similar in shape and scale to the scaled measurement vector.

**Figure 5:**
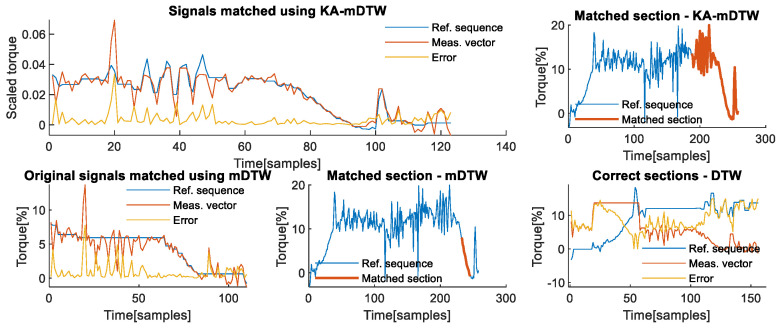
Matching of chosen signals from the 2nd axis. (**Upper left**) Optimally matched section of the reference sequence and the entire measurement vector and the sample-wise matching error. (**Upper right**) Reference sequence profile with the highlighted section, which was correctly interpreted and optimally matched with the measurement vector. (**Lower left**) Section of reference sequence incorrectly recognized and matched with measurement vector using mDTW without scaling the signals. (**Lower middle**) Reference sequence profile with the highlighted section, which was incorrectly recognized and matched with the measurement vector using mDTW. (**Lower right**) The section of the reference sequence identified by KA-mDTW is matched with the measurement vector using DTW algorithm to determine a realistic matching error.

The upper left part of Figure 5 shows how the KA-mDTW algorithm has successfully matched these two signals, i.e., recognized the correct section of the reference sequence that needs to be matched with the measurement vector, as shown in the upper right part of Figure 5, enabling correct interpretation of the physical process, i.e., contact task dynamics. The corresponding matching cumulative error when KA-mDTW was used is 0.4173. For comparison purposes, the diagram shown on the lower left part of Figure 5 shows how the mDTW algorithm would match the two signals if they were in their original form. As shown, the entire measurement vector would be matched with the very end of the reference sequence, corresponding to the load-releasing phase. This matching completely misses the phase when the robot was stationary just prior to releasing the load, thus not interpreting the task dynamics correctly. Due to the incorrect selection of the corresponding section of the reference sequence, as seen in the lower middle part of Figure 5, the cumulative matching error for this case when mDTW was used is deceivingly small, at 0.2199. A correct way to calculate the real error would be to use the classical DTW algorithm to optimally match the section of the reference sequence highlighted on the upper right part of Figure 4, which was chosen by the KA-mDTW algorithm, with measurement vector, without applying scaling by the lever lengths. In that case, the cumulative error of the matching shown on the lower right part of Figure 5, accounting for the different scales of signals, is 3.1506, which is drastically higher than that of KA-mDTW.

**Figure 6:**
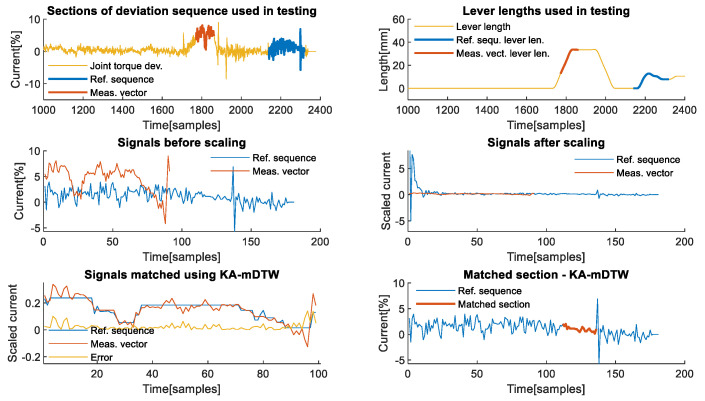
Analysis of example from the 4th axis. (**Upper left**) Deviation signal throughout the task with sections of the deviation signal used as reference sequence and the measurement vector. (**Upper right**) Sections of the lever lengths corresponding to the chosen reference sequence and the chosen measurement vector. (**Middle left**) Signal used as reference sequence and measurement vector in their original values prior to scaling by the algorithm. (**Middle right**) Signals used as reference sequence and measurement vector after scaling was applied. (**Lower left**) Graph shows optimally matched section of the reference sequence and the entire measurement vector as well as the sample-wise matching error. (**Lower right**) Reference sequence profile with the highlighted section, which was correctly interpreted and optimally matched with the measurement vector.

**Fourth axis analysis**: The analysis of the manipulation contact task on the fourth axis enables illustrating another aspect related to the proper setting of the reference sequence. This axis was unaffected by the manipulation of load during the first four pick and place movements due to zero equivalent lever length, but it is affected during the fifth and sixth movements due to changes in tool orientation, as shown in Figure 6 in the upper sections. The reference sequence in blue must be chosen to include exclusively non-zero lever lengths for the observed joint if there is any possibility that that joint will be affected by the contact force. Otherwise, the joint cannot be used for effective comparison with the measurement vector. The middle right part of Figure 6 shows that the scaled reference has some significant peaks at the beginning, which are caused by division with small values of equivalent lever length. If the lever length is equal to zero, the algorithm does not work properly, which is a property that needs to be addressed. In the case of the second axis, it was explained that the reference signal must include all phases of the contact task that may occur when there is a lever. If only certain phases of the contact task are performed while the lever length is different from zero, then the reference signal does not have to include the phases during which the lever length is equal to zero, only those during which it is over a certain small threshold. If there is a theoretical chance that a certain phase not included in the reference signal may occur, then the reference sequence is not representative enough and needs to be changed. In case there are mutually exclusive non-zero lever length phases, the reference sequence can be composed of two separately recorded reference sequences that combined include all possible non-zero lever phases. In the presented scenario, only transporting, lowering, and the releasing phase of the manipulation task were possible due to the robot’s configuration.

The lower left part of Figure 6 shows that the KA-mDTW algorithm can optimally match the reference sequence shown in blue, and the measurement vector shown in red with a cumulative error is 2.3941. The lower right part highlights in red the section of the reference to which the measurement vector was matched, which corresponds to the actual situation, although it is slightly shorter. For the same signals without scaling, the cumulative sum of error, compensated for the different scaling, is 19.2877, even with matching with the incorrect section of the reference subsequence. When DTW is applied to the correct section of the reference sequence, the realistic cumulative error is even higher, at 28.2313, showing the importance of the scaling phase of the KA-mDTW algorithm.

### 4.2. Consistent Tool Frame Direction of Force

As a representative for the type of contact forces, a snap-fit assembly task was chosen. It features a profile that can easily be mistaken for a collision due to its nature [31]. Therefore, the importance of proper interpretation of this signal is very high, as it may have serious consequences for production. The effects that this assembly contact task dynamics has on joint manifest themselves in the form of peaks in measurement values. With an aim to demonstrate the versatility of the algorithm in terms of signal availability using this example, instead of absolute values of currents, the current-based estimations of torque were used together with measurements of joint angles. The values of this signal were readily available as outputs from the Denso VP-6242 robot, and in this paper, they will be referred to interchangeably as torques or torque estimates.

**Figure 7:**
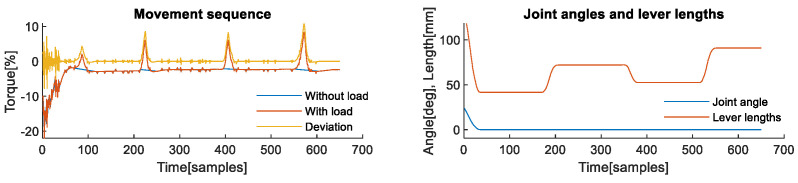
Signals obtained during snap-fit assembly task from the 1st axis. (**left**) Measurements of joint torques while the robot was performing the task without and with assembly of the object. The difference between them corresponds to the profile of the contact-task-induced deviation. (**right**) Measurement of the joint angle used to calculate equivalent lever lengths.

**First axis analysis**: The first axis is interesting for analysis, because it is usually not affected by gravity and is theoretically relatively insensitive to the influence of external forces. The latter is due to gearing ratios and powerful motors, which need to be used because of the high moments of inertia that it must withstand. The analysis of the effects that the dynamics of the snap-fit assembly contact task have on the first axis of the robot was performed using measurements and values presented in Figure 7. The deviation signal, shown in yellow on the left graph of Figure 7, which is used as input to KA-mDTW algorithm together with joint angles shown in blue on the right part of the same figure, has different dynamics compared to the corresponding signals analysed in the manipulation task examples. The reason is that during this task, the robot only moved to assume new positions for the assembly, while the force was exerted on the work object while the robot was stationary. This situation is present in various contact tasks of this type, and therefore, the conclusions made during this analysis are not limited to snap-fit assembly.

**Figure 8:**
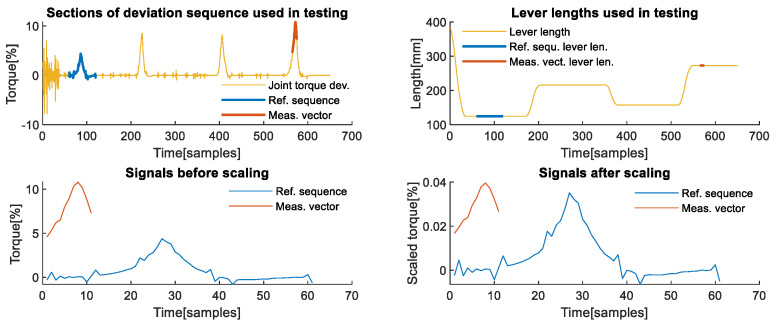
Matching chosen signals from the 1st axis. (**Upper left**) Sections of the deviation signal used as reference sequence and the measurement vector during analysis. (**Upper right**) Sections of the lever lengths corresponding to chosen testing signals. (**Lower left**) Signals used as reference sequence and measurement vector in their original values prior to scaling by the algorithm. (**Lower right**) Signals used as reference sequence and measurement vector after scaling was applied.

**Figure 9:**
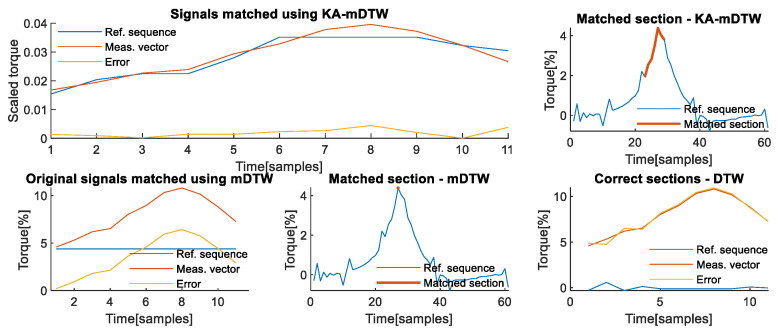
Matching of chosen signals from the 1st axis. (**Upper left**) Graph shows optimally matched section of the reference sequence and the entire measurement vector as well as the sample-wise matching error. (**Upper right**) Reference sequence profile with the highlighted section, which was optimally matched with the measurement vector. The highlighted section correctly interprets the phase of the contact task contained in the measurement vector. (**Lower left**) Section of reference sequence matched with measurement vector using mDTW without scaling the signals. A matching error has the same shape as the measurement vector, since the entire vector was only matched with a single point of reference, which was closest in value. (**Lower middle**) Reference sequence profile with the highlighted section, which is just a point matched with the measurement vector using mDTW. The highlighted section corresponds to the peak of the reference signal, since it was the closest to all the values within the measurement vector. (**Lower right**) The section of the reference sequence, which was optimally matched using KA-mDTW from the upper right graph, is matched with the measurement vector using DTW algorithm to determine a realistic matching error.

Similar to previous examples, Figure 8 in the upper left part shows sections of the deviation sequence, shown in yellow, which were used as reference sequence and measurement vector, highlighted in blue and red, respectively. The same is valid for lever lengths on the upper right graph of the same figure. From the lower left part of Figure 8, it is possible to observe that the selected reference sequence shown in blue and the measurement vector shown in red are similar only in shape but not in scale and values, although they originate from the same task. Scaling, which is an integral part of the KA-mDTW algorithm, makes these signals much easier to compare and interpret, as shown in the lower right part of Figure 9.

Figure 9 in the upper left part shows that the observed signals shown in blue and red were correctly matched, which is confirmed by the error signal shown in the yellow and cumulative matching error of 0.0207. The reference sequence segment corresponding to the measurement vector was correctly identified, as highlighted on the upper right part of the same figure, indicating that the contact task dynamics would be correctly interpreted. The most striking observation is that the mDTW algorithm is powerless in this situation and only manages to match the entire measurement with a single point from the reference sequence, which can be seen in the lower left and middle parts of Figure 9. Although striking, this result from mDTW is expected, and it is truly the best result it could theoretically achieve. Nevertheless, the error shown in yellow on the lower left graph has a cumulative value of 0.3106, which is significantly higher than with KA-mDTW. However, the biggest implication of this matching is that the contact force would be completely wrongly interpreted and would surely indicate some erroneous state. When the DTW algorithm is used to match the section of the reference sequence identified by the KA-mDTW with the measurement vector, the best result it can achieve is 0.7009.

**Figure 10:**
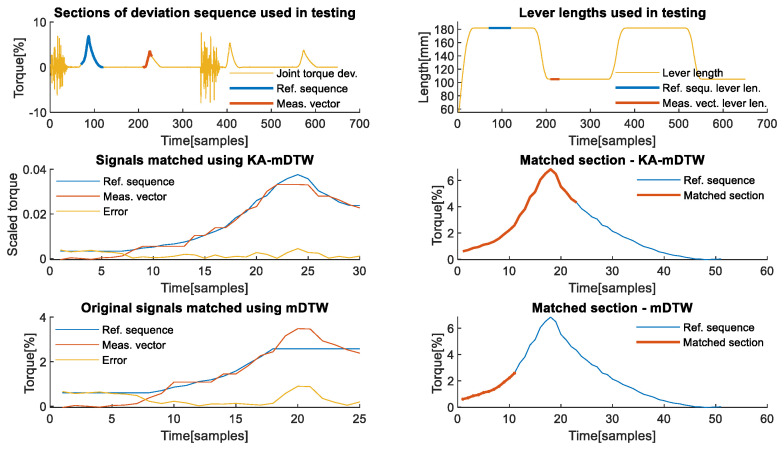
Matching chosen signals from the 3rd axis. (**Upper left**) Section of the deviation signal used as a reference sequence during analysis, and the section used as the measurement vector. (**Upper right**) Sections of the lever lengths corresponding to the chosen reference sequence and the chosen measurement vector. (**Middle left**) Optimally matched section of the reference sequence, the entire measurement vector, and the sample-wise matching error. (**Middle right**) Reference sequence profile with the highlighted section, which was correctly recognized, optimally matched with the measurement vector. (**Lower left**) Section of reference sequence matched with measurement vector using mDTW without scaling the signals and matching error. (**Lower right**).

**Third axis analysis**: The third axis of the robot is one of the most affected axes, regardless of the contact task type. The selected example aims to illustrate two things, the first of which can be observed from the upper graphs of Figure 10. In the upper left graph, the deviation signal shown in yellow on this axis shows that the first and the third deviation caused by the snap-fit contact task have similar intensity due to the identical corresponding equivalent lever lengths, which is also valid for the second and fourth deviation-induced peak.

The signals chosen for comparison as reference sequence and measurement vector, highlighted in blue and red, respectively, on the upper parts of Figure 10, demonstrate performance in the situation opposite of the one analysed for the first robot axis. In a situation when the reference signal, shown in blue on the middle right part of Figure 10, has higher values than the measurement vector shown in red, the performance of KA-mDTW algorithm is unaltered. As shown in the middle right part of the same figure, it correctly identifies the section of the reference sequence and optimally matches it with a cumulative matching error of 0.0518. The mDTW algorithm performs better than in the example from the first axis with a cumulative matching error of 0.0473, which is due to the incorrect section with which it was matched, leading to dynamics misinterpretation. The equivalent matching error calculated using DTW is 0.1640.

## 5. Conclusions

Contents of this paper address the issue of unmodelled dynamics related to contact tasks performed by industrial robots. The reasons for not considering contact task dynamics include modelling or implementation complexity, insufficient knowledge of the task, and underestimation of the effect they have. Consequences, however, are almost universally negative with regards to the reliability, observability, and predictability of the process involving them. From the perspectives of Industry 4.0 and 5.0, where collision erroneous state detection and handling, as well as operation context brokerage, are the basis of successful implementation, each of the mentioned drawbacks gains more significance.

This paper proposes to include the contact task dynamics implicitly by observing their effects on individual robot joints and understanding how this influence transforms with changes in the spatial relation of joints. Correct interpretation of the effects of contact forces enables the identification of phases of the task, and their comparison with expected values enables the detection of erroneous states.

The underlying idea presented in the second section of the paper proposes that the effects of contact task dynamics are observed through deviations of joint currents or torques from the nominal values. Samples from a representative task execution form a reference sequence with which all future samples of deviation will be compared. Since the effects that an external contact force may have on a robot joint change with changes in the spatial relation between the joints, so do the profiles of the detected deviation.

To enable comparison between different profiles of deviations, an equivalent lever length parameter is introduced based on the considerations of its correlation with the deviation signal. The equivalent lever length is calculated for each joint at each sample based on the DH parameters of the robot and measurements of the joint angles and used to scale the corresponding sample of joint current or torque. The sample-wise scaling of samples of the reference sequence and the measurement vector enables their reliable comparison using a modification of Dynamic Time Warping (mDTW), a previously developed algorithm for real-time matching of signals with content and sampling differences.

Fusion of equivalent lever lengths scaling and mDTW resulted in the newly proposed Kinematically Augmented mDTW (KA-mDTW). This versatile and efficient algorithm introduces robot kinematics into flexible time series matching. Furthermore, the proposed changes influence the rules for calculation of the d matrix of mDTW algorithm, based on which the matching surface is shaped and optimally matched pairs are determined.

The KA-mDTW algorithm was thoroughly tested using real measurements from an industrial robot performing load manipulation and snap-fit assembly tasks as representative examples from two groups of contact tasks. For each example, the efficiency of the algorithm was demonstrated and commented on with regards to the cumulative matching error compared to the mDTW algorithm, as well as the correct identification of the matched section of the reference sequence. Due to its inherent signal scaling ability, KA-mDTW’s cumulative matching error was shown to be drastically lower than the equivalent error of mDTW. Correctly matched sections of the reference sequence enable proper interpretation of the phases of the contact task dynamics, as well as reliable detections of erroneous states.

The testing phase also commented that the reference sequence must be representative of the contact task, including all possible effects that the task may have on the joint. These include all phases of the task during which the equivalent lever length is non-zero. That effectively means that the algorithm can match detected deviation successfully only if the reference sequence includes an example of the same phase of the task that caused the deviation. It was also noted that references recorded with small lever lengths cause peaks due to scaling with small values. For the same reason, the reference sequence must not include samples during which the equivalent lever length was zero.

The proposed approach and the algorithm successfully avoided implementation issues by relying on versatile automatic parameter identification and processing automation. However, the fact that the algorithm’s performance depends on setting the proper reference sequence leaves room for user-induced performance reduction. Further research will examine algorithms for evaluation of the reference sequence and information they include. Another direction will focus on predicting time intervals when the deviation is expected, i.e., the intentional contact of the robot and its surroundings.

## Data Availability

Not applicable.
